# Editorial: Protecting the welfare of individuals operating in organized sport

**DOI:** 10.3389/fspor.2025.1580898

**Published:** 2025-03-12

**Authors:** James L. Rumbold, James A. Newman, Andrew J. Higham, Louise Davis, Ashley Stirling

**Affiliations:** ^1^ School of Sport and Physical Activity, College of Health, Wellbeing and Life Sciences, Sheffield Hallam University, Sheffield, United Kingdom; ^2^ Department of Psychology, School of Social Sciences, Heriot-Watt University, Edinburgh, United Kingdom; ^3^ Department of Psychology, Umeå School of Sport Sciences, Umeå University, Umeå, Sweden; ^4^ Faculty of Kinesiology and Physical Education, University of Toronto, Toronto, ON, Canada

**Keywords:** abuse, concussion management, duty of care, interpersonal violence, mental health, safeguarding, well-being, welfare

**Editorial on the Research Topic**
Protecting the welfare of individuals operating in organized sport

## 1 Introduction

The issue of physical and mental welfare in sport has gained increasing prominence in recent years, leading various personnel (e.g., the media, professional bodies and researchers) to consider aspects such as safeguarding, clean sport, overtraining, and the wider ethics surrounding the duty of care of those involved in this environment. Given the importance of these aspects, it is unsurprising that researchers as well as professional bodies and sports organizations have tasked themselves with protecting individuals in sport from the harmful effects of wrongdoing and factors which might create significant injury risk (e.g., concussion). As a result, numerous positive advances have been made to understand and improve the welfare and well-being of those in sport, as well as to raise awareness and education at the micro- (e.g., peer-to-peer), meso- (e.g., coach-athlete, coach-parent, parent-athlete), exo- (e.g., professional leagues, national governing bodies), and macro-system (e.g., media and societal views) levels. Despite such positive advances, they have not always been well communicated between system levels of sport or across international perspectives. Therefore, this special topic sought to address these concerns, utilizing various international perspectives to provide recommendations to protect individuals across the sporting systems.

## 2 Contents of the research topic

In this Frontiers research topic, it is pleasing to see a number of multinational collaborative studies, with our topic including research findings from Canada, the United States of America, and eight European countries. We believe this multinational collaboration illustrates an international research commitment to better understanding and addressing safeguarding and welfare in organized sport. The authors who have contributed to this research topic have utilized a range of methods (e.g., mini reviews, quantitative, qualitative, and intervention methods) to expand our knowledge of how sporting personnel, organizations, and national governing bodies can protect the welfare of various individuals who are involved in organized sport. Below, we provide a research topic summary of author contributions based on three overarching themes: (1) Abuse, bullying, interpersonal violence and maltreatment studies; (2) Exploring mental health in elite athletes, and; (3) Parent and match official perspectives on concussion management.

### 2.1 Abuse, bullying, interpersonal violence, and maltreatment studies

#### 2.1.1 Reviews

Gillard et al. (2024) conducted a mini review to synthesize knowledge regarding the roles, readiness to change and training needs of athlete health and performance team members to handle interpersonal violence in sport. From 43 articles that were reviewed, it was identified that very little research has directly assessed athlete health and performance team members’ needs to facilitate safety and eradicate interpersonal violence in sport. The authors offer a series of expert recommendations to guide future research and practice.

#### 2.1.2 Quantitative findings

Muhonen et al. (2024) surveyed Finish elite and leisure athletes to ascertain whether there is any correlation between emotional abuse, athletic identity, and disclosure of abusive behaviors. Results indicated that a salient athletic identity was related to a higher prevalence of emotional abuse, children were most susceptible to emotional abuse, and both salient athletic identity and emotional abuse negatively predicted athletes' disclosure of emotionally abusive coaching practices.

To develop a tool to measure coaches’ beliefs regarding the effectiveness of interpersonal violence practices in sport, Parent et al. (2024) consulted with an expert panel of six researchers, and carried out item reduction to arrive at a 25-item PIEVS scale containing six dimensions. Following this, the authors conducted an initial validation of the PIEVS scale with 690 coaches to determine the 1-factor solution for both the 25-item, and a 9-item short form version of the scale. In addition, convergent and divergent validity was achieved by identifying significant relationships with disempowering and (inversely) empowering motivational climates.

Vveinhardt and Kaspare (2024) surveyed 371 Lithuanian Kyokushin karate athletes to measure bullying experiences and signs of stress, anxiety and depression. The findings indicated that 75.5% of Kyokushin karate athletes had experienced unethical behavior by their coaches or others at least once. In addition, signs of stress, anxiety and depression were found to be correlated with damage in the areas of communication, social relations and physical health.

The work of Willson et al. (2024) examined the relationship between psychological abuse, athlete satisfaction, eating disorder and self-harm indicators in current and retired Canadian national team athletes using a maltreatment survey. The results indicated a negative correlation between psychological abuse and athlete satisfaction, and a positive correlation with eating disorders and self-harm indicators.

#### 2.1.3 Qualitative findings

Adams et al. (2024) implemented interviews to explore how and why former intercollegiate athletes identified their head coach as emotionally abusive. The athletes’ narratives suggested that a coach is labelled abusive if they diminished performance, neglected holistic development, were inconsistent, provided negative emotional responses, and dehumanized athletes.

Newman and Rumbold (2024) conducted interviews with safeguarding and welfare personnel in English professional and semi-professional football to explore their understanding of maltreatment. Findings indicated that wrongdoing in football contexts is nuanced in comparison to other sports, as certain forms of maltreatment are driven by the unique nature of football environments. This work provides a platform for practitioners and researchers to raise awareness of maltreatment in professional football whilst also challenging the prevailing workplace culture.

#### 2.1.4 Intervention mapping

The work of Adriaens et al. (2024) implemented an intervention mapping approach as a guiding framework to systematically develop a bystander training program (i.e., Safe Sport Allies), to train youth sport participants and youth sport coaches to act as effective bystanders. The authors propose a variety of behavior change program principles to improve sport participants’ bystander behaviors.

### 2.2 Exploring mental health in elite athletes

Work by Küttel et al. (2024) interviewed seven Danish international elite athletes to unveil perspectives on career and mental health development, whilst considering the dynamic interplay of personal and environmental factors. Findings highlighted the complex interplay of factors affecting mental health, and emphasize the need for creating supportive environments that help athletes manage the intense demands of elite sport.

To explore the media coverage relating to German elite athletes’ mental illness, Hapig et al. (2024) conducted a systematic search and screening of eleven German newspapers and magazines. Through synthesizing more than a decade's worth of German print media, it was concluded that there is an enhanced awareness towards the topic of mental illness and those affected in recent years. This was evidenced by the increased integration of responsible reporting elements, the inclusion of diversified perspectives, and the considerate selection of content.

### 2.3 Exploring parent and match official perspectives on concussion management

Hagopian et al. (2024) conducted two focus groups with 11 parents in Canada to gain their perspectives and experiences with Neuropsychological Baseline Testing (NBT) to better manage concussion injuries. Using inductive content analysis, some common themes included navigating uncertainty about the nature of concussion and its management process, and mixed NBT reviews regarding its usefulness in concussion management.

In the concluding article, Jorgensen et al. (2024) conducted semi-structured interviews to investigate match officials’ perspectives and experiences regarding sport-related concussion management and the Blue Card protocol (i.e., the removal of athletes from play if they are suspected to have sustained a concussion) in community rugby in Canada. The authors highlighted that despite potential benefits to athlete welfare, the welfare of match officials is risked due to sporting cultures that tolerate abuse.

